# Vitamin C—Protective Role in Oxidative Stress Conditions Induced in Human Normal Colon Cells by Label-Free Raman Spectroscopy and Imaging

**DOI:** 10.3390/ijms22136928

**Published:** 2021-06-28

**Authors:** Karolina Beton, Beata Brozek-Pluska

**Affiliations:** Laboratory of Laser Molecular Spectroscopy, Institute of Applied Radiation Chemistry, Lodz University of Technology, Wroblewskiego 15, 93-590 Lodz, Poland; karolina.beton@dokt.p.lodz.pl

**Keywords:** colon cancer, Raman spectroscopy, Raman imaging, biomarkers, biomedical imaging, cell culture, supplementation, vitamin C

## Abstract

Colorectal cancer is the second most frequently diagnosed cancer worldwide. Conventional diagnostics methods of colorectal cancer can detect it at an advanced stage. Spectroscopic methods, including Raman spectroscopy and imaging, are becoming more and more popular in medical applications, and allow fast, precise, and unambiguous differentiation of healthy and cancerous samples. The most important advantage of Raman spectroscopy is the ability to identify biomarkers that help in the differentiation of healthy and cancerous cells based on biochemistry of sample and spectra typical for lipids, proteins, and DNA. The aim of the study was to evaluate the biochemical and structural features of human colon cell lines based on Raman spectroscopy and imaging: normal cells CCD-18 Co, normal cells CCD-18 Co under oxidative stress conditions, and normal cells CCD-18 Co at first treated by using tert-Butyl hydroperoxide and then supplemented by vitamin C in high concentration to show the protective role of vitamin C in micromolar concentrations against ROS (Reactive Oxygen Species). Raman data obtained for normal cells injured by ROS were compared with spectra typical for cancerous cells. Statistically assisted analysis has shown that normal ROS-injured and cancerous human colon cells can be distinguished based on their unique vibrational properties. The research carried out proves that label-free Raman spectroscopy may play an important role in clinical diagnostics differentiation of normal and cancerous colon cells and may be a source of intraoperative information supporting histopathological analysis.

## 1. Introduction

Colorectal cancer (CRC), the third most common cancer in men and the second in women worldwide, is the third leading cause of death in the world. The mortality rate from this type of cancer is approximately 60% in the United States and Europe [1]. CRC is characterized by high metastasis and poor prognosis [2,3,4]. 

In general, the risk factors for CRC can be divided into three main groups:

(1) environmental (e.g., high-fat diet, high-calorie diet, diet low in silage, vegetables, and fruit);
(2) internal (e.g., adenomas, ulcers, Crohn’s syndrome);
(3) genetic (e.g., familial adenomatous polyposis) [5].

A total of 75 to 95% of CRC cases occur in people without any genetic load [6,7]. Those with a family load for two or more first-degree relatives (such as a parent or sibling) have a two- to three-fold greater risk of suffering CRC [8,9]. 

The additional risk factors influencing the development of CRC are largely related to aging, male sex [7], and lifestyle including a high intake of fat, sugar, alcohol [10], red meat, processed meats, obesity, smoking, and a lack of or insufficient physical activity [6,11,12].

Basically, cancer development is a complex multi-stage process that begins anywhere in the body as cells transform from normal to pathological. These changes may occur on their own or they may be induced by the presence or coexistence of factors that we divide into three main groups:

- physical, such as ultraviolet and ionizing radiation;
- chemicals, such as asbestos, tobacco smoke, aflatoxins and arsenic;
- biological, e.g., infections due to viruses, bacteria, or parasites.

In the first stage of CRC development, healthy cells in the lining of the colon or rectum change and grow and divide uncontrollably to form a mass called a tumor. Both genetic and environmental factors can change the dynamics of this process. CRC most often begins with a polyp, a non-cancerous growth that can develop on the inner wall of the colon or rectum with increasing age and then can transform into cancer or metastatic cancer [13].

Each day, the content of the human colon can be described as a diverse mix of bile, mucus, gut microflora, fermentation products, unabsorbed food, and products of metabolism, including toxins, mutagens, and dissolved gases. In such an environment, the mucosa of the colon is constantly exposed to dietary oxidants and a variety of bacteria. Permanent exposure of the mucosa and the organism itself to unfavorable conditions may lead to uncontrolled oxidative stress and DNA damage, which may lead to the development of cancer disease. Almost 95% of CRC are glandular carcinomas, while the remaining 5% are squamous, mixed, or undifferentiated cell types [1].

The protocols to prevent the occurrence and development of CRC include tests ranging from fecal occult blood tests (iFOBT, gFOBT), blood test, colonoscopy examination of high-risk individuals to detect and remove precancerous lesions using biopsy, molecular research, and drug chemotherapy [9,14]. One of the most popular diagnostic imaging methods for CRC is colonoscopy. The supporting techniques are colonography (virtual colonoscopy) or X-ray examination. Unfortunately, the colonoscopy does not provide answers about the origin of tissue abnormality. The suspicious lesion must be removed and further analyzed by a trained pathologist using the gold standard staining protocols that are particularly time consuming, subjective, and reliant on highly skilled personnel. Moreover, colonic perforation occurs in approximately 1 in 1000 cases of colonoscopy; hemorrhagic complications may also occur during or after examination. That is why the development of new diagnostics methods including those based on Raman spectroscopy and imaging stimulate so many research groups worldwide.

The first in vivo Raman measurements of human gastrointestinal tissue were published in 2000 by Shim et al. [15]. This study has shown that fiber-optic-coupled Raman spectroscopy can be successfully used for disease classification during in vivo measurements. FT-Raman studies of CRC have also been published by Andrade et al. [16]. The authors described a diagnostic algorithm useful to establish the spectral differences of the complex colon tissues to find characteristic Raman features. The first Raman and CARS (coherent anti-Stokes Raman scattering spectroscopy) data of colon tissue were published by Krafft et al. [17]. Comparison between CARS and spontaneous Raman images confirmed that results obtained using both methods are comparable, but the time needed for CARS maps acquisition is three orders of magnitude shorter. In 2013, Gerwert et al. [18] showed that the auto-fluorescence of colon tissue overlaps spatially with the fluorescence of antibodies against p53, which are of interest in routine immunohistochemistry in pathology analysis and indicates nuclei with mutated p53 of cancer cells. They have also shown many advantages of VIS region (532 nm) excitation compared to excitations of wavelengths from the IR region (785 or 830 nm) most often used in spectroscopic experiments [18].

Despite the continuous development of synthetic anticancer drugs, there is still a need to discover and research natural compounds with a therapeutic effect on the incidence and progression of cancer [19]. Vitamin C (ascorbic acid) is an essential micronutrient that must be provided in the diet or as a supplement. People have lost the ability to synthesize it naturally due to a mutation in the gene encoding the final enzyme in its biosynthetic pathway [20]. Vitamin C plays a significant role in many processes as a cofactor of enzymes involved in processes important for cancer development: antioxidant defense, transcription, and epigenetic regulation of gene expression. Vitamin C has also been shown to have a beneficial effect on the immune system by mobilizing it to fight infections, and prevents inflammation, which is key in the body’s fight against cancer cells [21]. The anti-cancer potential of vitamin C is suggested by the results of many laboratory studies in animals and cell cultures [22,23,24,25,26,27,28,29,30]. In anti-cancer research, not only is vitamin C used, but also its derivatives, including compounds with increased lipophilicity and resistance to oxidation [31].

In principle, the antioxidative action of vitamin C takes place by direct scavenging of ROS, especially peroxides and peroxynitrite [32]. Moreover, in the work of Diaz et al. [33], it has been shown that ascorbate can revitalize alpha-tocopherol, which in turn helps prevent lipid oxidation in the body. On the other hand, without ascorbate, the alpha-tocopheroxyl radical may play a pro-oxidative role and continue or even enhance the lipid peroxidation process. By acting as an electron donor, vitamin C can also reduce superoxide anions, hydroxyl radicals, singlet oxygen, and hypochlorous acid generated during metabolic respiration, wherein ATP is produced. Vitamin C can therefore protect against mutations caused by oxidative DNA damage, lipid peroxidation and the oxidation of amino acid residues in order to maintain protein integrity by inhibiting the emission of free radicals [34].

The level of vitamin C in human plasma is tightly regulated to maintain a physiological concentration of about 50 to 70 µM, which limits its ability to be highly concentrated in cells just by oral ingestion. The tight regulation of vitamin C homeostasis is primarily controlled by four regulatory systems [35]:

- intestinal absorption,
- accumulation and distribution in tissues,
- degree of use and recyclability for reuse,
- renal excretion and reabsorption.

This can be accomplished through a variety of mechanisms, including passive diffusion, facilitated diffusion, active transport, and re-use through a reabsorption system [36].

For the above reasons, scientific research on antioxidant delivery conducted using the intravenous method, omitting the so-called first-pass effect associated with oral supplementation, has gained popularity [28,29,37,38,39].

The aim of the study was to evaluate the biochemical features of human colon cell lines based on label-free Raman spectroscopy and imaging: normal CCD-18 Co, normal CCD-18 Co under oxidative stress conditions, normal CCD-18 Co at first treated by using tert-Butyl hydroperoxide and then supplemented by vitamin C in micromolar concentration to show the protective role of vitamin C against ROS. Data attained for normal cells injured by ROS were compared and contrasted with data typical for cancerous cells CaCo-2.

Statistically assisted analysis of Raman data shows that normal, ROS-injured and cancerous human cells of human colon can be distinguished based on their unique vibrational properties typical for proteins, lipids, and DNA.

The research carried out prove that label-free Raman spectroscopy may play an important role in clinical diagnostics and in the future may be a source of intraoperative information supporting histopathological analysis in the differentiation of healthy and cancer cells.

## 2. Results

In this section, data obtained by Raman spectroscopy and imaging for human colon cells both before and after generation of ROS, including supplementation with vitamin C, are expounded. We also present a comparison of spectra for normal human colon cells (CCD-18 Co), for normal colon cells under oxidative stress conditions and for the cancerous human colon cell line (CaCo-2).

In general, the Raman vibrational spectra consists of two interesting regions: Raman fingerprint region: 500–1800 cm^−1^ and high frequency region: 2700–3100 cm^−1^ (region 1800-2700 cm^−1^ is excluded from consideration due to the lack of Raman bands).

To properly rise to biochemical changes in both normal and cancerous human colon cell lines by Raman spectroscopy and imaging, we will closely inquire how the Raman-based method responds to generated ROS. The experiments will extend our knowledge of the protective effect of antioxidants and veritable influence of the ROS generation on cancer development.

**Figure 1:**
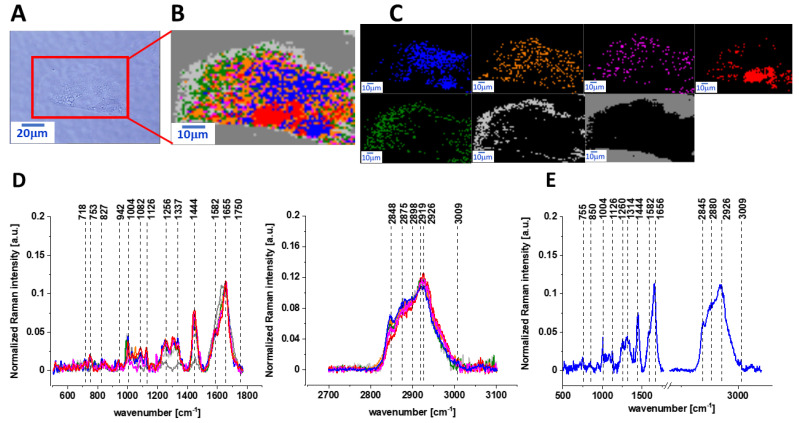
The microscopy image (**A**), Raman image (**B**) of human colon normal single cell CCD-18 Co constructed based on cluster analysis (CA) method; Raman images of all clusters identified by CA assigned to lipid-rich regions (blue and orange), mitochondria (magenta), nucleus (red), cytoplasm (green), cell membrane (light grey), and cell environment (dark grey) (**C**); the average Raman spectra typical for CCD-18 Co human normal colon cell for all identified clusters for low frequency and high frequency region (**D**), and the average Raman spectrum for human normal colon cells—for cells as a whole (**E**), all data for experiments performed without any supplementation, cells measured in PBS, colors of the spectra correspond to the colors of clusters; excitation laser line 532 nm; number of cells = 6.

Figure 1 presents the microscopy image, Raman image of human colon normal single cell CCD-18 Co constructed based on cluster analysis (CA) method, Raman images of all clusters identified by CA assigned to lipid-rich regions, mitochondria, nucleus, cytoplasm, cell membrane, and cell environment, the average Raman spectra typical for CCD-18 Co human normal colon cell for all identified clusters for low frequency and high frequency region, and the average Raman spectrum for human normal colon cells—for cells as a whole, all data for the experiments were performed without any supplementation, the cells were measured in PBS (Phosphate Buffered Saline), and the colors of the spectra correspond to the colors of clusters.

As we have mentioned above, ROS are produced by living organisms as a result of natural cellular metabolism, but in such a case, the concentrations of them have low to moderate functions in physiological cell processes; at high ROS concentrations, adverse modifications to cell components, such as lipids, proteins, and DNA, can be noticed [40]. The shift in balance between oxidant/antioxidant in favor of oxidants seems to be crucial for understanding many dysfunctions of human cells including cancer transformation. However, before we start the analysis of ROS-treated human normal colon cells, let us focus on CCD-18 Co cells supplemented with vitamin C in micromolar concentration to check how this vitamin influences the cells’ vibrational features.

**Figure 2:**
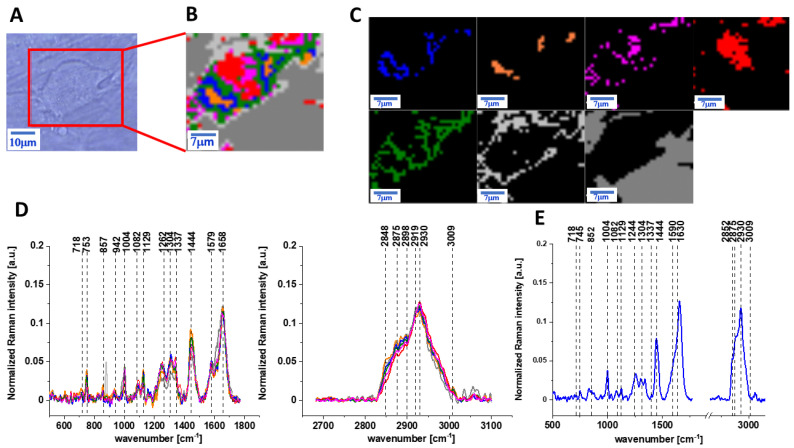
The microscopy image (**A**), Raman image (**B**) of human colon normal single cell CCD-18 Co after 24 h of vitamin C supplementation constructed based on cluster analysis (CA) method, Raman images of all clusters identified by CA assigned to: lipid-rich regions (blue and orange), mitochondria (magenta), nucleus (red), cytoplasm (green), cell membrane (light grey), and cell environment (dark grey) (**C**), the average Raman spectra typical for CCD-18 Co human normal colon cell for all identified clusters for low frequency and high frequency region (**D**), and the average Raman spectrum for human normal colon cells—for cells as a whole (**E**), cells measured in PBS, colors of the spectra correspond to the colors of clusters; excitation laser line 532 nm; number of cells = 6.

Figure 2 presents the microscopy image, Raman image of human colon normal single cell CCD-18 Co after 24 h of vitamin C supplementation constructed based on the Cluster Analysis (CA) method; Raman images of all clusters identified by CA assigned to lipid-rich regions, mitochondria, nucleus, cytoplasm, cell membrane, and cell environment; the average Raman spectra typical for CCD-18 Co human normal colon cell for all identified clusters for low frequency and high frequency region, and the average Raman spectrum for human normal colon cells—for cells as a whole, all data for the experiments were performed with supplementation with vitamin C of 50 µM concentration in medium; the cells were measured in PBS; the colors of the spectra correspond to the colors of clusters.

**Figure 3:**
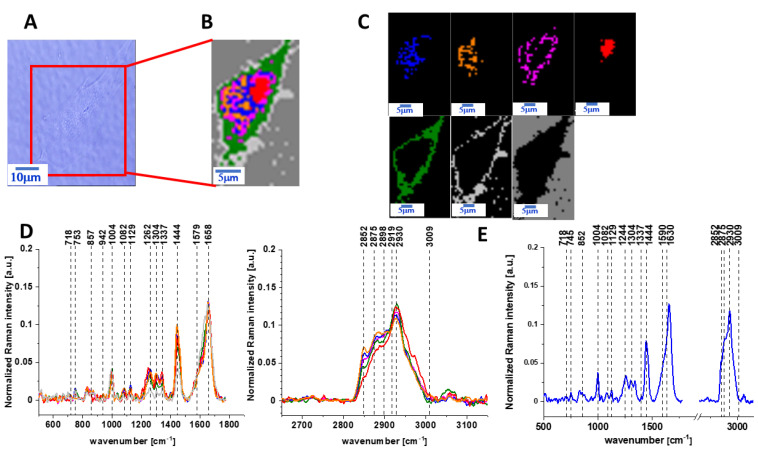
The microscopy image (**A**), Raman image (**B**) of human colon normal single cell CCD-18 Co after 48 h of vitamin C supplementation constructed based on cluster analysis (CA) method; Raman images of all clusters identified by CA assigned to lipid-rich regions (blue and orange), mitochondria (magenta), nucleus (red), cytoplasm (green), cell membrane (light grey), and cell environment (dark grey) (**C**); the average Raman spectra typical for CCD-18 Co human normal colon cell for all identified clusters for low frequency and high frequency region (**D**), and the average Raman spectrum for human normal colon cells—for cells as a whole (**E**), cells measured in PBS, colors of the spectra correspond to the colors of clusters; excitation laser line 532 nm; number of cells = 6.

The same Raman analysis was also performed for human normal colon cells CCD-18 Co with supplementation by vitamin C of 50 µM concentration in medium for an incubation time of 48 h. Figure 3 shows the microscopy image, Raman image of human colon normal single cell CCD-18 Co after 48 h of vitamin C supplementation constructed based on the cluster analysis (CA) method; Raman images of all clusters identified by CA assigned to lipid-rich regions, mitochondria, nucleus, cytoplasm, cell membrane, and cell environment; the average Raman spectra typical for CCD-18 Co human normal colon cell for all identified clusters for low frequency and high frequency region, and the average Raman spectrum for human normal colon cells—for cells as a whole, all data for the experiments were performed with supplementation with vitamin C of 50 µM concentration in medium; the cells were measured in PBS; the colors of the spectra correspond to the colors of the clusters.

The next step of our experiments was the analysis of Raman features typical for human colon normal cells CCD-18 Co under oxidative stress conditions generated by using tBuOOH with a concentration of 50 µM in medium for 24 and 48 h.

**Figure 4:**
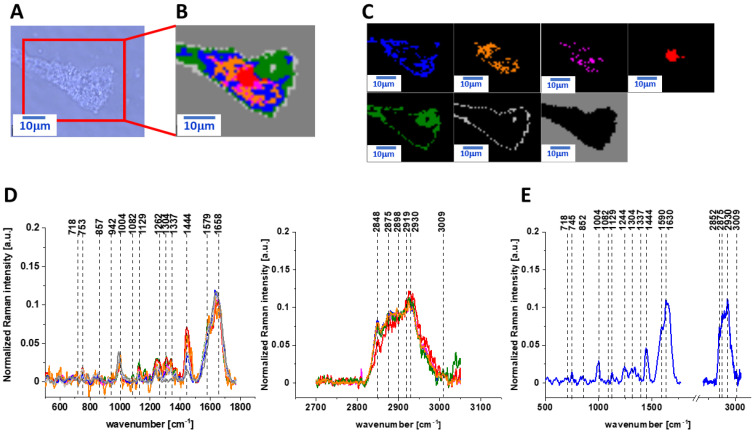
The microscopy image (**A**), Raman image (**B**) of human colon normal single cell CCD-18 Co under oxidative stress conditions generated by using tBuOOH for incubation time of 24 h and concentration of 50 µM in medium constructed based on cluster analysis (CA) method; Raman images of all clusters identified by CA assigned to lipid-rich regions (blue and orange), mitochondria (magenta), nucleus (red), cytoplasm (green), cell membrane (light grey), and cell environment (dark grey) (**C**); the average Raman spectra typical for CCD-18 Co human normal colon cell for all identified clusters for low frequency and high frequency region (**D**), and the average Raman spectrum for human normal colon cells - for cells as a whole (**E**), cells measured in PBS, colors of the spectra correspond to the colors of clusters; excitation laser line 532 nm; number of cells = 6.

**Figure 5:**
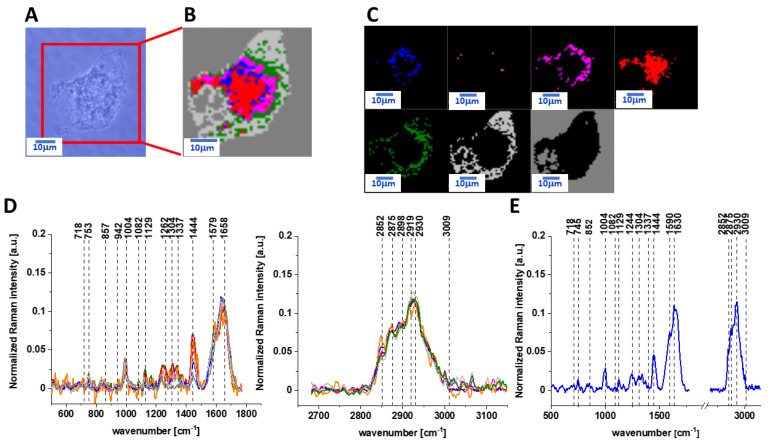
The microscopy image (**A**), Raman image (**B**) of human colon normal single cell CCD-18 Co under oxidative stress conditions generated by using tBuOOH for incubation time of 48 h and concentration 50 µM in medium constructed based on cluster analysis (CA) method, Raman images of all clusters identified by CA assigned to: lipid-rich regions (blue and orange), mitochondria (magenta), nucleus (red), cytoplasm (green), cell membrane (light grey), and cell environment (dark grey) (**C**), the average Raman spectra typical for CCD-18 Co human normal colon cell for all identified clusters for low frequency and high frequency region (**D**), and the average Raman spectrum for human normal colon cells—for cells as a whole (**E**), cells measured in PBS, colors of the spectra correspond to the colors of clusters, excitation laser line 532 nm, number of cells = 6.

Figure 4 and Figure 5 show the microscopy images, Raman images of human colon normal single cells CCD-18 Co under oxidative stress conditions generated by using tBuOOH on concentration of 50 µM in medium for 24 and 48 h, respectively.

Next, to check if the vitamin C shows the protective properties against ROS, we have measured human normal colon cells CCD-18 Co supplemented simultaneously by tBuOOH and vitamin C.

**Figure 6:**
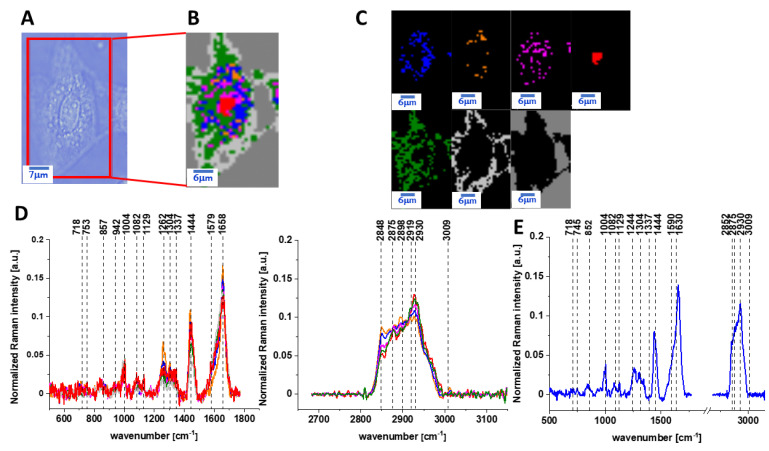
The microscopy image (**A**), Raman image (**B**) of human colon normal single cell CCD-18 Co for supplementation by tBuOOH (50 µM in medium) and vitamin C (50 µM in medium) for incubation time of 24 h constructed based on cluster analysis (CA) method; Raman images of all clusters identified by CA assigned to lipid-rich regions (blue and orange), mitochondria (magenta), nucleus (red), cytoplasm (green), cell membrane (light grey), and cell environment (dark grey) (**C**); the average Raman spectra typical for CCD-18 Co human normal colon cell for all identified clusters for low frequency and high frequency region (**D**), and the average Raman spectrum for human normal colon cells—for cells as a whole (**E**), cells measured in PBS, colors of the spectra correspond to the colors of clusters; excitation laser line 532 nm; number of cells = 6.

**Figure 7:**
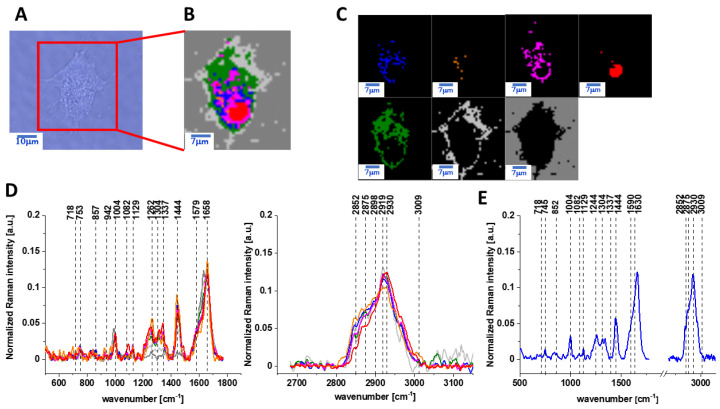
The microscopy image (**A**), Raman image (**B**) of human colon normal single cell CCD-18 Co for supplementation by tBuOOH (50 µM in medium) and vitamin C (50 µM in medium) for incubation time of 48 h constructed based on cluster analysis (CA) method; Raman images of all clusters identified by CA assigned to lipid-rich regions (blue and orange), mitochondria (magenta), nucleus (red), cytoplasm (green), cell membrane (light grey), and cell environment (dark grey) (**C**); the average Raman spectra typical for CCD-18 Co human normal colon cell for all identified clusters for low frequency and high frequency region (**D**), and the average Raman spectrum for human normal colon cells—for cells as a whole (**E**), cells measured in PBS, colors of the spectra correspond to the colors of clusters; excitation laser line 532 nm; number of cells = 6.

Figure 6 and Figure 7 show the vibrational data obtained for human normal cells CCD-18 Co for experiments performed with adding of tBuOOH and vitamin C for 24 or 48 h by Raman spectroscopy and imaging.

Table 1 featured the main chemical constituents that can be identified based on their vibrational properties in analyzed CCD-18 Co human, normal colon cells in normal conditions, in oxidative stress conditions generated by adding tBuOOH (discussed later in the manuscript) and for the experiments for vitamin C supplementation.

The main goal of the research undertaken in this study was the biochemical analysis of human normal colon cell line in normal or ROS conditions, demonstrating the antioxidant properties of vitamin C and comparative analysis of normal human colon cells in different conditions with human cancerous colon cells CaCo-2 based on the vibrational spectra of colon cells using label-free Raman spectroscopy and imaging. Figure S1 shows Raman spectra and imaging for cancerous CaCo-2 cells measured in PBS (see Supplementary Materials).

## 3. Discussion

Having reached this point, we can start the comparative analysis of spectroscopic data.

The Raman data for human colon cancer cells CaCo-2 and the comparison for normal human colon cells CCD-18 Co and human colon cancer cells CaCo-2 for experiments without any supplementation are shown in the Supplementary Materials (please see Figures S1 and S2).

At first, we compare the vibrational data for control sample—CCD-18 Co cells and normal cells after vitamin C supplementation. 

**Figure 8:**
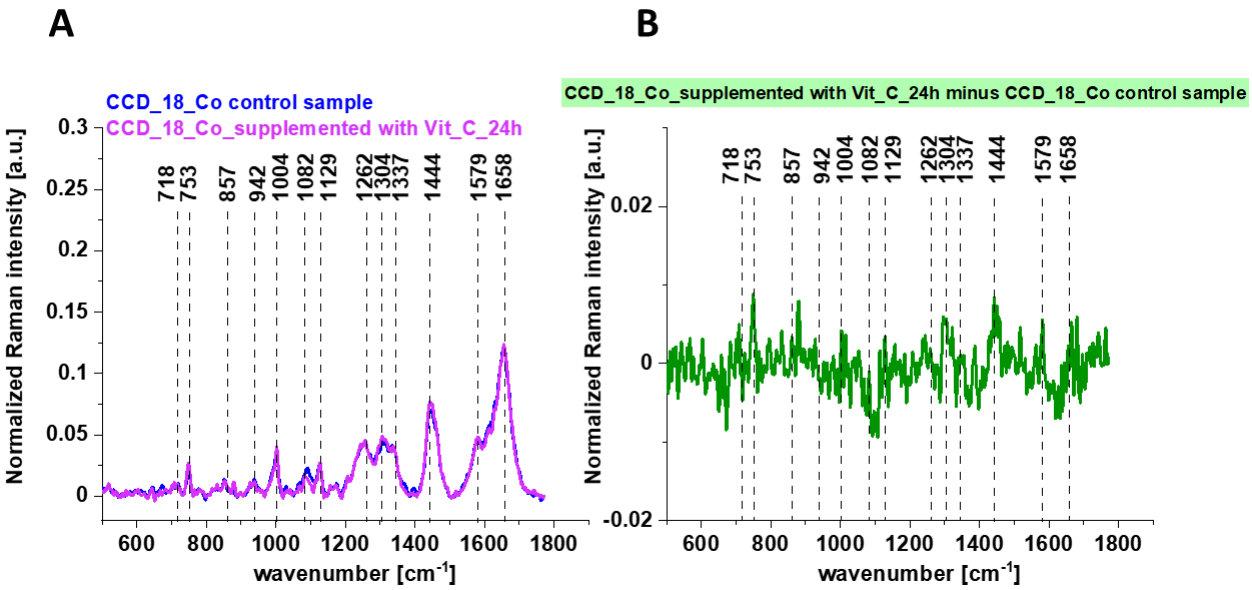
The average Raman spectra obtained for CCD-18 Co human normal control cells (control sample), the average Raman spectra typical for CCD-18 Co human normal control cells supplemented with vitamin C for 24 h (**A**) and the differential Raman spectrum (**B**) calculated based on spectra presented on panel (A); excitation laser line 532 nm; number of cells = 6 for each type.

Figure 8 shows the Raman mean spectra determined for cells as a single cluster for CCD-18 Co human normal control cells (control sample), with the addition of vitamin C (A) and the differential spectra calculated on their basis (B).

One can see from Figure 8 that the influence of vitamin C on Raman spectra typical for CCD-18 Co human colon normal cells is very subtle (the same result was obtained for 48 h vitamin C supplementation). This observation allows to link up all differences observed for experiments performed by using tBuOOH with ROS generation and their concentration modulated by vitamin C.

**Figure 9:**
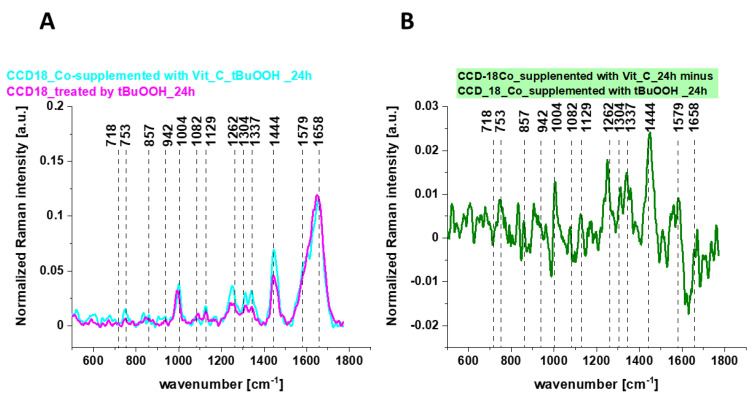
The average Raman spectra obtained for CCD-18 Co human normal colon cells in oxidative stress condition, the average Raman spectra typical for CCD-18 Co human normal colon cells supplemented with vitamin C and tBuOOH for 24 h (**A**) and the differential Raman spectrum (**B**) calculated based on spectra presented on panel (A); excitation laser line 532 nm, number of cells = 6 for each type.

Figure 9 shows the average Raman spectra of CCD-18 Co human normal colon cells in oxidative stress conditions generated by using tBuOOH and for samples supplemented simultaneously by tBuOOH and vitamin C (A) and the differential spectrum calculated from them (B).

Having reached this point, when the analysis based on Raman vibrational spectra for different human normal colon CCD-18 Co cells groups has been performed, we can comprehensively compare the results shown in Figure 1, Figure 2, Figure 3, Figure 4, Figure 5, Figure 6 and Figure 7.

Considering that CCD-18 Co human normal colon cells are basically composed of three types of macromolecules: proteins, nucleic acids, and lipids, in order to explore characteristic changes under ROS conditions and for cells supplemented with vitamin C, the qualitative and quantitative comparison between paired bands assigned to those classes of compounds will be discussed according to the biological attribution of them.

**Figure 10:**
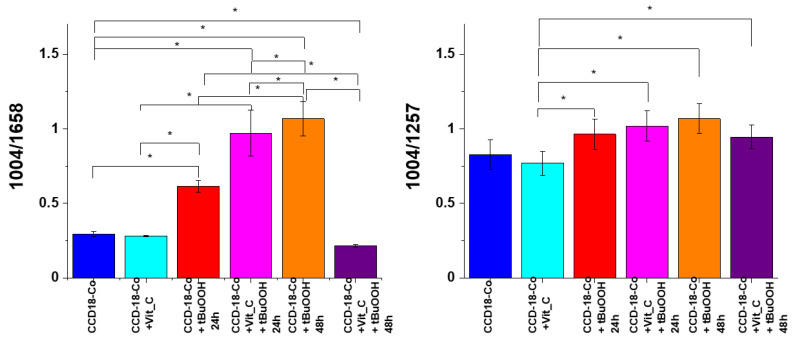
Raman band intensities ratios for selected Raman bands corresponding to proteins for 6 groups of normal human colon cells CCD-18 Co: control group (labeled CCD-18-Co, blue), group supplemented with vitamin C (labeled CCD-18 Co+Vit_C, turquoise), group supplemented with tBuOOH for 24h (labelled CCD-18 Co+tBuOOH, 24h, red), group supplemented with vitamin C and tBuOOH for 24h (labelled CCD-18 Co+Vit C+tBuOOH, 24h, magenta), group supplemented with tBuOOH for 48h (labelled CCD-18 Co+tBuOOH, 48h, orange), group. supplemented with vitamin C and tBuOOH for 48h (labelled CCD-18 Co +Vit C+tBuOOH, 48h, violet), the statistically significant results, based on ANOVA analysis, have been marked with asterix.

Figure 10 shows an analysis of the influence of oxidative stress generating factor—tBuOOH and antioxidant in the form of vitamin C on analyzed biological systems based on the composition of the basic building blocks of each cell in the form of proteins, DNA, and lipids. Bands 1257, 1658 cm^−1^ describe the protein content in cells, respectively: amide I and amide III bands, bands 750 and 1078 cm^−1^ reflect the amount of nucleic acids present in cells [43]; bands 1444, 2854 and 3009 cm^−1^ are characteristic for lipids [41].

As can be seen in Figure 10, for normal human colon CCD-18 Co cells, the addition of vitamin C does not affect the protein content, while the addition of tBuOOH—ROS generating agent—modulates their amount. As the intensity of the 1004 cm^−1^ band is practically constant after the addition of vitamin C compared to non-supplemented cells, the effect of increasing the value of the ratio 1004/1658 for the CCD-18 Co + tBuOOH system confirms the decrease of intensity of the 1658 cm^−1^ band, and the modification the amount of proteins after cell supplementation with an ROS-generating compound. The results for 24 and 48 h supplementation showed a similar tendency, although the protective effect of antioxidant in the form of vitamin C turned out to be stronger for 48 h.

In the case of proteins, high activity of free oxygen and nitrogen radicals leads to the formation of oxidized protein products. The damage generated concerns both polypeptide chains as well as amino acid residues. The peroxidation of polypeptide chains resembles the peroxidation of lipids except for the chain nature of the observed changes [44]. The hydroxyl radical initiates the oxidation of the protein chain by cleavage of the hydrogen atom on the α-amino acid carbon. The resulting alkyl radical reacts with oxygen to generate the product in the form of an alkylhydroperoxide. Alkylhydroperoxide can transform into an alkoxy radical, which in turn is responsible for activating the fragmentation of polypeptide chains. Breakage of the polypeptide chain can also occur as a result of free radical oxidation of aspartate, glutamate, and proline. In proteins, apart from the fragmentation of the chain, as a result of ROS, the oxidation of amino acid residues, including aromatic amino acid residues, may also occur. The oxidation of amino acids with a free amino, amide or hydroxyl group leads to the formation of carbonyl derivatives present primarily in amino acid derivatives in the form of aldehydes or ketones. Modification of cysteine residues may result in breaking disulfide bonds and breaking down the tertiary structure of the protein [45].

**Figure 11:**
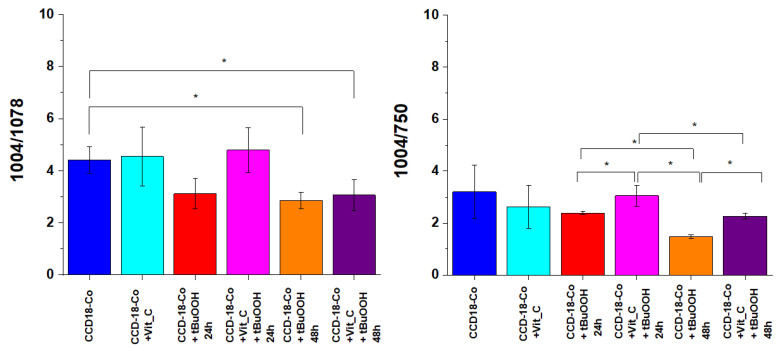
Raman band intensities ratios for selected Raman bands corresponding to nucleic acids for six groups of normal human colon cells CCD-18 Co: control group (labeled CCD-18 Co, blue), group supplemented with vitamin C (labeled CCD-18 Co+Vit_C, turquoise), group supplemented with tBuOOH for 24 h (labelled CCD-18 Co+tBuOOH, 24 h, red), group supplemented with vitamin C and tBuOOH for 24 h (labelled CCD-18 Co+Vit C+tBuOOH, 24 h, magenta), group supplemented with tBuOOH for 48 h (labelled CCD-18 Co + tBuOOH, 48 h, orange), group supplemented with vitamin C and tBuOOH for 48 h (labelled CCD-18 Co +Vit C + tBuOOH, 48 h, violet); the statistically significant results, based on ANOVA analysis, have been marked with asterix.

Figure 11 presents analogous analysis performed based on Raman bands typical for nucleic acid.

As shown in Figure 11, for normal human colon cells CCD-18 Co, the addition of vitamin C does not affect the content of nucleic acids (within the standard deviation); however, the addition of tBuOOH—ROS generating agent caused a change in the values of the analyzed Raman band intensity ratios. The addition of tBuOOH resulted in the decrease of the intensity of the bands typical of nucleic acids for both 24 and 48 h of supplementation. Nucleic acids are much more stable than proteins and, when exposed to ROS, do not easily transform into a free-radical state (damage is usually quickly repaired; damaged bases are “excised” and excreted from cells). In general, reactions of the hydroxide radical with nucleic acids damage the base sugar residues and also result in the breaking of phosphodiester bonds. Thymidine, from which thymidine peroxides are formed, is particularly susceptible to the action of radicals. The product of DNA oxygen damage is 8-hydroxy-deoxyguanosine (8-OhdG). Increased levels of 8-OhdG have been shown in the course of many diseases, including cancer, diabetes, and chronic inflammation [46].

**Figure 12:**
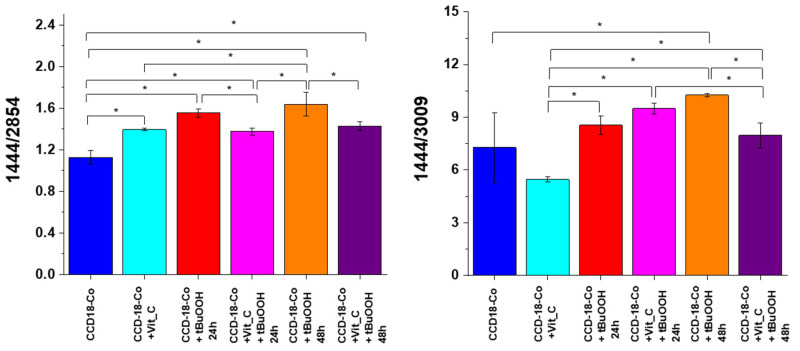
Raman band intensities ratios for selected Raman bands corresponding to lipids for six groups of normal human colon cells CCD-18 Co: control group (labeled CCD-18 Co, blue), group supplemented with vitamin C (labeled CCD-18 Co+Vit_C, turquoise), group supplemented with tBuOOH for 24 h (labelled CCD-18 Co + tBuOOH, 24 h, red), group supplemented with vitamin C and tBuOOH for 24 h (labelled CCD-18 Co+Vit C + tBuOOH, 24 h, magenta), group supplemented with tBuOOH for 48 h (labelled CCD-18 Co + tBuOOH, 48 h, orange), group supplemented with vitamin C and tBuOOH for 48 h (labelled CCD-18 Co +Vit C + tBuOOH, 48 h, violet). As shown in Figure 12, it also affects the lipids’ presence in cells of the human colon; the statistically significant results, based on ANOVA analysis, have been marked with asterix.

Figure 12 shows the analysis based on the bands characteristic for lipids.

Lipid peroxidation under the influence of ROS consists of the oxidation of polyunsaturated fatty acid residues, which are numerous in phospholipids, and this process takes place in a non-enzymatic way and as a result of enzymatic reactions. The product of the initiation step characteristic of lipid peroxidation are free alkyl radicals, which then react with oxygen in prolongation reactions to form free peroxide radicals. These radicals, located at the end of the system of double bonds, react with subsequent molecules of polyunsaturated fatty acids to give peroxides (hydroperoxides) of fatty acids, which are the first products of relative stability. The termination step involves reactions directly between the radicals formed, leading to the formation of fatty acid dimers and keto or hydroxy fatty acids. The enzymatic lipid peroxidation leads to the formation of lipid hydroperoxides with the participation of enzymes from the lipoxygenases group. Enzymatic lipid peroxidation differs from non-enzymatic lipid peroxidation in that during the process with the participation of enzymes, the generated lipid peroxide radicals are converted into anions, as a result of which the substrate containing an unpaired electron is depleted, and thus, the generation of hydroperoxides is inhibited. Enzymatic lipid peroxidation is also an initiating process for the formation of biologically active compounds such as prostaglandins, thromboxanes, and leukotrienes. In contrast, arachidonic acid released by phospholipase A2 is the starting compound in the synthesis involving cyclooxygenases. Importantly, the products of lipid oxidation may react with proteins and DNA, contributing to their further damage [47]. The analysis of the intensity ratios of Raman bands 1444/2854 and 1444/3009 confirms the destructive effect of tBuOOH on lipids and the “protective” nature of vitamin C both for 24 and 48 h supplementation.

To confirm the ability of the presented Ramana spectra to differentiate the samples of CCD-18 Co in conditions under consideration—ROS-injured cells and vitamin C supplementation in a non-speculative way, which has practical value—we have also performed the multivariate principal component analysis (see Supplementary Materials, Figure S3).

Finally, we have also compared the results obtained for normal human colon cells with results typical for cancer cells CaCo-2. Table 2 presents results of spectroscopic data analysis.

One can see from Table 2 that the intensities of Raman band ratios typical for proteins, nucleic acids, and lipids depend on experimental conditions and the type of human colon cells. Comparison of data obtained for normal and cancerous cells confirm that CaCo-2 cells contain more proteins and nucleic acids and less lipids (including unsaturated fraction). Comparison of data obtained for normal cells in oxidative stress conditions and cancerous type shows that the oxidation generated by adding tBuOOH is effective and data for cells injured by ROS are comparable to that obtained for cancerous cells CaCo-2.

## 4. Materials and Methods

### 4.1. Cell lines and Cell Culture

CCD-18 Co cell line (ATCC^®^ CRL-1459™) was purchased from ATCC: The Global Bioresource Center. CCD-18 Co cell line was cultured using ATCC-formulated Eagle’s minimum essential medium with L-glutamine (catalog No. 30-2003). To make the complete growth medium, fetal bovine serum was added to a final concentration of 10%. Every two to three days, a new medium was used. The cells obtained from the patient are normal myofibroblasts in the colon. The biological safety of the CCD-18 Co cell line has been classified by the American Biosafety Association (ABSA) as level 1 (BSL-1). The CaCo-2 cell line was also purchased from ATCC and cultured according to the ATCC protocols. The CaCo-2 cell line was obtained from a patient—a 72-year-old Caucasian male diagnosed with colon adenocarcinoma. The biological safety of the obtained material is classified as level 1 (BSL-1). To make the medium complete, Eagle’s minimum essential medium with L-glutamine had fetal bovine serum added to it to a final concentration of 20%. The medium was renewed once or twice a week.

### 4.2. Cultivation Conditions

The cell lines (CCD-18 Co, Caco-2) used in the experiments in this study were grown in flat-bottom culture flasks made of polystyrene with a cell growth surface of 75 cm^2^. Flasks containing cells were stored in an incubator providing environmental conditions at 37 °C, 5% CO_2_, 95% air.

### 4.3. Cell Treatment with Vitamin C and/or tBuOOH

Cells used for research were seeded onto CaF_2_ windows (25 × 1 mm) at a low density of 10^4^ cells/cm^3^. After 24 h incubation on the CaF_2_, standard growth medium was removed, and vitamin C solution was added for 24 or 48 h. For the stress agent variant, tBuOOH at a concentration of 50 µM with or without 50 µM of vitamin C was added. After 24 or 48 h, the cells were rinsed with phosphate-buffered saline (PBS, Gibco, 10010023, pH 7.4 at 25 °C, 0.01 M) to remove any residual medium and an excess vitamin C that did not penetrate inside the cells. Furthermore, PBS was removed, and cells were fixed in paraformaldehyde (4% buffered formaldehyde) for 10 min and washed once more with PBS. The Raman confocal measurements were made immediately after the preparation of the samples.

All the stress compound solutions, based on tBuOOH, were prepared by pre-diluting in the PBS solvent. After obtaining the initial concentration of 20,000 μM, it was diluted in a medium intended for a given type of cell, so as to obtain the concentration of 200 μM and finally 50 μM.

### 4.4. Raman Spectroscopy and Imaging

All maps and Raman spectra presented and discussed in this paper were recorded using the confocal microscope Alpha 300 RSA+ (WITec, Ulm, Germany) equipped with an Olympus microscope integrated with a fiber with a 50-µm core diameter with a UHTS spectrometer (Ultra High Through Spectrometer) and a CCD Andor Newton DU970NUVB-353 camera operating in default mode at −60 °C in full vertical binning mode. A 532-nm excitation laser line, which is the second harmonic of the Nd: YAG laser, was focused on the sample through a Nikon objective lens with magnification of 40x and a numerical aperture (NA = 1.0) intended for cell measurements performed by immersion in PBS. The average excitation power of the laser during the experiments was 10 mW, with an integration time of 0.5 s for Raman measurements for the high frequency region and 1.0 s for the low frequency region. An edge filter was used to filter out the Rayleigh scattered light. A piezoelectric table was applied to set the test sample in the right place by manipulating the XYZ positions and consequently record Raman images. Spectra were acquired with one acquisition per pixel and a diffraction grating of 1200 lines/mm. Cosmic rays were removed from each Raman spectrum (model: filter size: 2, dynamic factor: 10) and the Savitzky–Golay method was implemented for the smoothing procedure (order: 4, derivative: 0). All data was collected and processed using a special original software WITec Project Plus. All imaging data were analyzed by cluster analysis (CA), which allows for the grouping of a set of vibrational spectra that bear resemblance to each other. CA was executed using WITec Project Plus software with a centroid model and k-means algorithm, in which each cluster is represented by one vector of the mean. Data normalization was performed using the model divided by norm, which was wrought with the Origin software serving as a mathematical and statistical analysis tool.

### 4.5. Chemical Compounds

L-Ascorbic acid, reagent grade, crystalline catalogue number A7506-25G, Luperox^®^ TBH70X, tert-Butyl hydroperoxide solution catalogue number 458139, bisBenzimide H 33342 trihydrochloride catalogue number B2261, and Red Oil-O catalogue number O0625 were purchased from Sigma-Aldrich and used without additional purification. An XTT (2,3-Bis-(2-Methoxy-4-Nitro-5-Sulfophenyl)-2*H*-Tetrazolium-5-Carboxanilide) proliferation kit with catalogue number 20-300-1000 was purchased from Biological Industries.

### 4.6. XTT Cells Viability Tests

The XTT test is grounded on the cleaving of the yellow tetrazolium salt XTT to form an orange water-soluble formazan product by activity of dehydrogenase enzyme in the active mitochondria. The quantity of formazan product is directly proportional to the number of living and respiring cells. Diminution in the amount of living cells results in a depletion in the overall activity of mitochondrial dehydrogenases in the sample. This decrease is directly in consonance with the amount of generated orange formazan, monitored by the measurement of absorbance using a multi-sensing microplate spectrophotometer. With the XTT test, cell proliferation and viability of the cells after treatment with the test item are determined colorimetrically. The use of the XTT reagent consists of the statistical calculation of the metabolic activity of cells wherewithal a colorimetric technique in response to changed environmental conditions in which they are located.

The XTT test requires the use of a 450 nm wavelength, from which the specific signal of the sample is obtained, and 650 nm, which is the referential sample during the test. XTT reagent is used to compute the activity of metabolic processes in living cells. Tetrazolium salts are converted in cells by means of special enzymes to formazan; however, this reaction takes place properly only in cells with an undamaged metabolism. XTT is believed to be excluded from entering cells due to a negative net charge [48]. Scientific work suggests that the reduction of the XTT dye transpires at the cell surface, which facilitates the transport of electrons across the cytoplasmic membrane. Mitochondrial oxidoreductases are believed to contribute significantly to the response to the XTT reagent and their reducers are transferred to the plasmalemma. Ultimately, it was found that XTT assays actually measure the redox state of pyridine nucleotide cells [48,49]. XTT tests on cell lines were carried out on a multi-detecting BioTek Synergy HT model reader designed for microplate testing. The test protocol was created especially for the presented experiments.

**Scheme 1:**
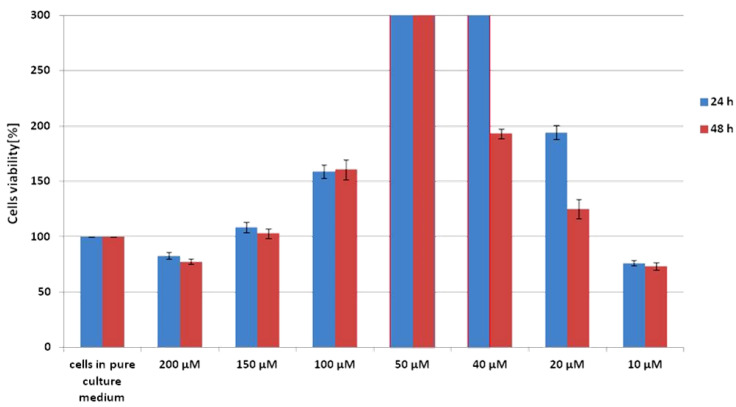
Results of XTT comparison of the percent viability for CCD-18 Co human normal colon cells supplemented with different concentrations of vitamin C at two different time intervals with the standard deviation ± SD.

Scheme 1 shows the results of the XTT test obtained for CCD-18 Co human normal colon cells supplemented with vitamin C in various concentrations and in various time intervals.

The addition of vitamin C at different concentrations to cells with a normal structure does not cause damage to the cells, which can be concluded from the fact that their viability rate is higher than for the referential sample. For the highest and the lowest concentration of vitamin C, a slight decrease in survival below 100% can be observed. The attached data show that the most optimal concentration for this cell line, the action of which stimulate the metabolic processes of cells, is 50 μM. The basis for such a conclusion is the percentage of cell viability, which, for this concentration, is above the measuring range of the device, allows us to conclude that cells in such an environment grow and multiply more often and faster. This result is identical for incubation times of 24 and 48 h, so it can be suspected that maintaining a constant saturation of colon cells with vitamin C at a concentration of 50 μM supports the proper proliferation and vital functions of cells.

### 4.7. Statistical Analysis

All results regarding the analysis of the intensity of the Raman spectra as a function of the type and time supplementation are presented as the mean ± SD, *p* < 0.05, where:

SD—standard deviation,

*p*—probability value.

ANOVA analysis (analysis of variance) was conducted using Origin software (significance level—0.05, range test—Tukey). 

PCA analysis were performed using MATLAB (MathWorks, USA) with PLS-Toolbox (EigenvectorResearch Inc., USA).

## 5. Conclusions

In the presented paper, we conducted an investigation into the cell culture of human colon cells using Raman spectroscopy, which shows that this spectroscopic technique is a useful tool that enables the detection of cancerous changes in biological material in an unambiguous manner. Moreover, spectroscopy and Raman imaging do not have a destructive effect on the tested biological material, so that the sample can be subjected to further analysis at a selected time.

Raman spectroscopy and imaging have been successfully applied to characterize and differentiate normal and cancerous cell lines from the human colon based on the unique vibrational ‘fingerprint’ of the sample. Moreover, single-cell substructures such as the nucleus, lipid-rich regions, mitochondria, plasma membrane and cellular cytoplasm can be visualized on the basis of Raman spectra using the cluster analysis algorithm, which allows for grouping vibrational spectra and the creation of clusters classifying individual cell areas.

Raman studies of human colon cells also show that vibrational spectroscopy can provide results that differentiate between normal and cancerous cells in the human colon based on the basic building blocks of the cells. The biochemical composition of normal cells and cancerous cells are different. The content of DNA, proteins, and lipids, including the fraction of unsaturated fatty acids, differentiates healthy and cancerous samples. The analysis of the intensity of the lipids/proteins/nucleic acid bands shows that these classes of compounds can classify the analyzed samples into normal and pathological ones.

Analysis of vibrational spectra for supplemented cells also showed that the antioxidant properties of vitamin C can be demonstrated using Raman spectroscopy and imaging based on bands characteristic of DNA, proteins, and lipids.

Based on Raman band intensities attributed to proteins, nucleus acids, and lipids, as well as ratios 1004/1658, 1004/1257, 1004/1078, 1004/750, 1444/3009, 1444/2854 calculated based on them, we have confirmed the protective role of vitamin C for cells in oxidative stress conditions for a label-free and nondestructive spectroscopic Raman method.

## Figures and Tables

**Table 1:** Band positions and tentative assignments for human normal colon cells from control sample, in oxidative stress conditions generated by adding tBuOOH (discussed later in the manuscript) and for the experiments for vitamin C supplementation. Data based on the average Raman spectra for cells as a whole measured in PBS, excitation laser line 532 nm [41,42].

Wavelenght [cm^−1^]	Tentative Assignments
**755**	Nucleic acids, DNA, Tryptophan, Nucleoproteins
**850**	Tyrosine
**1004**	Phenyloalanine
**1126**	Saturated fatty acids, cytochrome C
**1260**	Amide III (C–N stretching + N–H bending)
**1314**	CH_3_CH_2_ twisting mode of collagen
**1444**	Lipids and Proteins
**1582**	CN_2_ scissoring and NH_2_ rocking of mitochondria and phosphorylated proteins
**1656**	Amide I (C=O stretch)
**2848**	Lipids, fatty acids
**2880**	Lipids and proteins
**2931**	Due primarily to protein
**3009**	(=C-H), lipids, fatty acids

**Table 2:** Raman band intensities ratios for selected Raman bands corresponding to proteins, nucleic acids, and lipids for human normal colon cells CCD-18 Co in normal conditions, in oxidative stress conditions generated by adding tBuOOH and for cancerous human colon cells CaCo-2.

	CCD-18 Co	CCD-18 Co + tBuOOH		CaCo-2
		24 h	48 h	
**1004/1658**	0.29 ± 0.02	0.61 ± 0.04	1.06 ± 0.10	0.18 ± 0.03
**1004/1257**	0.82 ± 0.17	0.96 ± 0.13	1.07 ± 0.10	0.52 ± 0.02
**1004/1078**	4.41 ± 0.50	3.12 ± 0.50	2.86 ± 0.30	1.55 ± 0.30
**1004/750**	3.19 ± 1.00	2.87 ± 0.05	1.47 ± 0.07	1.88 ± 0.50
**1444/2854**	1.12 ± 0.06	1.55 ± 0.03	1.64 ± 0.10	1.49 ± 0.04
**1444/3009**	7.27 ± 1.99	8.55 ± 0.53	10.25 ± 0.08	8.95 ± 1.1

## Data Availability

The raw data underlying the results presented in the study are available from Lodz University of Technology Institutional Data Access for researchers who meet the criteria for access. Request for access to those data should be addressed to the Director of the Institute of Applied Radiation Chemistry, Lodz University of Technology. Data requests might be sent by email to the secretary of the Institute of Applied Radiation Chemistry: mitr@mitr.p.lodz.pl.

## Supplementary Materials

The following are available online at https://www.mdpi.com/article/10.3390/ijms22136928/s1.
