# Supplementary material for: Vitamin C—Protective Role in Oxidative Stress Conditions Induced in Human Normal Colon Cells by Label-Free Raman Spectroscopy and Imaging
Source: Int J Mol Sci. 2021 Jun 28;22(13):6928. doi: 10.3390/ijms22136928 (PMC8267733; doi:10.3390/ijms22136928)
Supplement: Supplementary file 1 [file ijms-22-06928-s001.zip › ijms-1212719-supplementary.pdf]

SUPPLEMENTARY MATERIALS

Figure S1 shows the microscopy image, Raman image of human colon cancer single cell CaCo-2 constructed based on Cluster Analysis (CA) method, Raman images of all clusters identified by CA assigned to: lipid-rich regions (blue and orange), mitochondria (magenta), nucleus (red), cytoplasm (green), cell membrane (light grey), and cell environment (dark grey) (C), the average Raman spectra typical for CaCo-2 human cancer colon cell for all identified clusters for low frequency and high frequency region (D), and the average Raman spectrum for human cancer colon cells - for cells as a whole (E), cells measured in PBS, colors of the spectra correspond to the colors of clusters, excitation laser line 532 nm, number of cells=6.

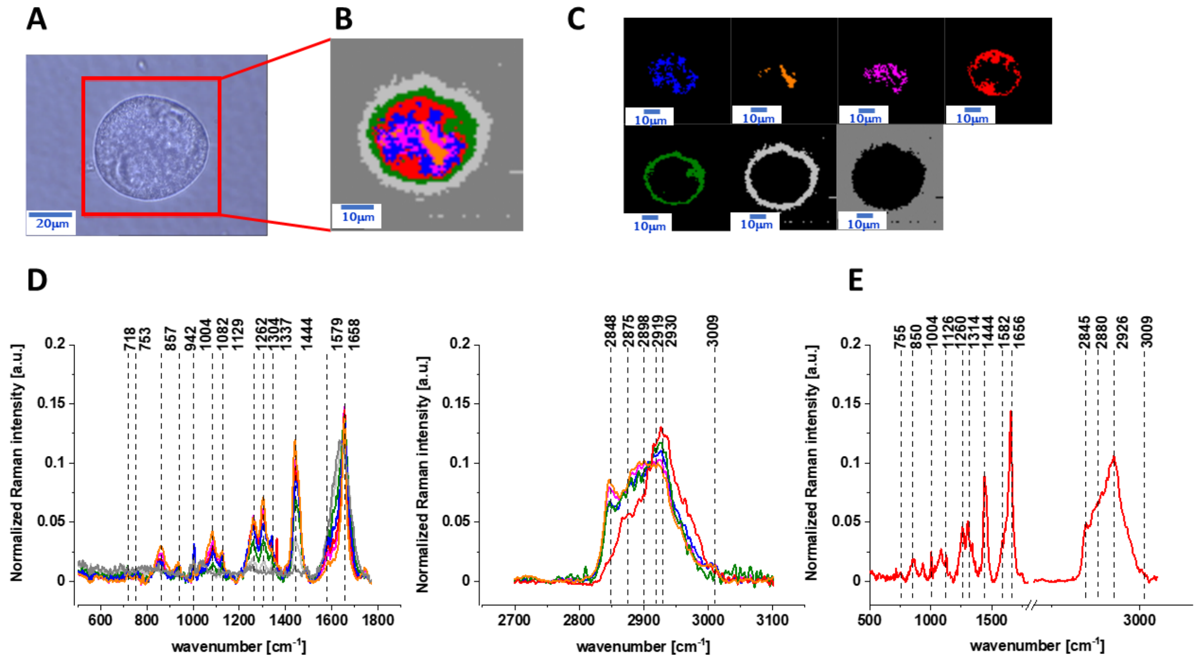

Figure S1. The microscopy image (A), Raman image (B) of human colon cancer single cell CaCo-2 constructed based on Cluster Analysis (CA) method, Raman images of all clusters identified by CA assigned to: lipid-rich regions (blue and orange), mitochondria (magenta), nucleus (red), cytoplasm (green), cell membrane (light grey), and cell environment (dark grey) (C), the average Raman spectra typical for CaCo-2 human cancer colon cell for all identified clusters for low frequency and high frequency region (D), and the average Raman spectrum for human cancer colon cells - for cells as a whole (E), cells measured in PBS, colors of the spectra correspond to the colors of clusters, excitation laser line 532 nm, number of cells=6.

Figure S2 presents the differential spectrum of normal and cancerous human colon cells (marked in violet color), the mean spectrum +/- SD (SD-Standard Deviation) typical for the human cancerous colon cell line (CaCo-2, marked in red), and the mean spectrum +/- SD typical for the human normal colon cell line (CCD-18 Co, marked in blue), generated using Cluster analysis (CA) for cells as a single cluster, cells measured in PBS, excitation laser line 532 nm, number of cells = 3.

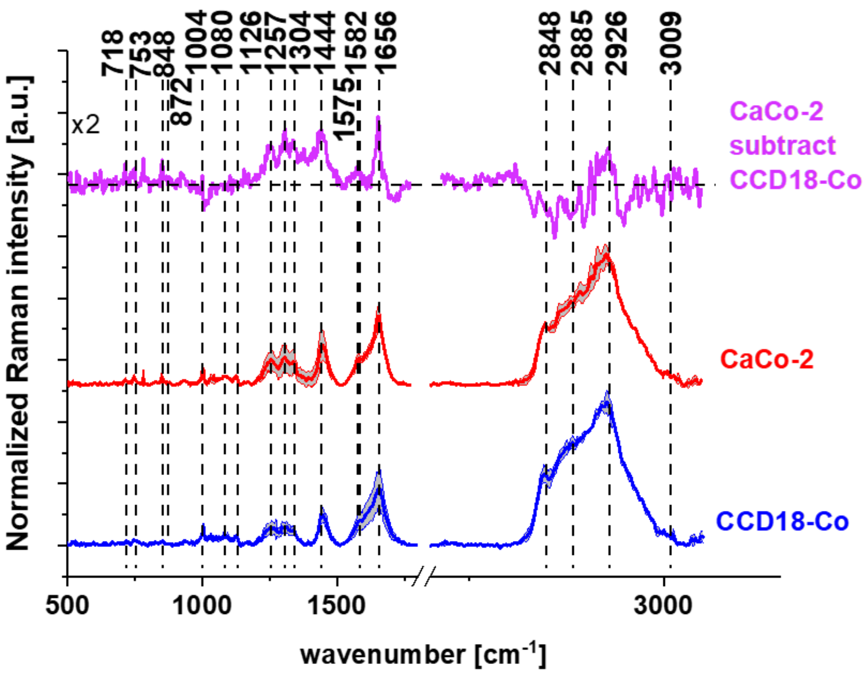

Figure S2. The differential spectrum of normal and cancerous human colon cells (marked in violet color), the mean spectrum +/- SD (SD-Standard Deviation) typical for the human cancerous colon cell line (CaCo-2, marked in red), and the mean spectrum +/- SD typical for the human normal colon cell line (CCD-18 Co, marked in blue), generated using Cluster analysis (CA) for cells as a single cluster, number of cells = 3 [42].

The analysis of the differential spectrum presented in Figure S2 shows that the most significant differences between normal and cancerous cells for human colon are observed for the following frequencies: 753, 872, 1082, 1575, 1004, 1585 and 2926 cm^{-1}, which according to literature reports can be assigned to individual cell components such as: DNA, RNA, lipids, proteins or unsaturated fatty acids.

The Amide III (1230-1300 cm^{-1}) and Amide I (1600-1690 cm^{-1}) bands are widely used to study the secondary structure of proteins and to estimate the total amount of proteins in a test sample. The analysis of Figure S2 shows that these bands are positive on the differential spectrum, which confirms the higher amount of proteins in the biochemical composition of the Caco-2 cell line presenting cancerous cells. The bands found at 1004, 1585 and 2926 cm^{-1} are associated with proteins and also show higher intensity in cancer cells. The same tendency is observed for RNA/DNA peaks (750, 1078 cm^{-1}). More and more studies indicate the potential use of cell-free DNA as a biomarker of prefatory symptoms in cancer diagnosis, prognosis and monitoring. A similar relationship as for DNA and RNA can be observed for the band at 848 cm^{-1}, which is attributed to mono- and disaccharides. This effect can be explained by higher concentrations of glycolysis intermediates such as acetates and lactates. All the phosphate-associated bands observed around 753, 872, 1082, 1585 cm^{-1} also show a greater proportion in the pathologically altered Caco-2 cell line. The literature has shown a higher level of phosphorylation of cancerous tissues in many organs, including the breast, brain and colon. A negative correlation for the colon cancer cell line Caco-2 in Figure S2 can be observed for lipid nature high frequency bands (bands appearing in the region of 2845-2875 cm^{-1}),

which confirms the different lipid metabolism in normal and cancerous cells.

Figure S3 shows the scores 3D plot obtained from PCA (Principle Component Analysis) in two different projections to confirm the ability of Raman spectroscopic data to differentiate 6 groups of human normal colon cells: control group (blue square), group supplemented with vitamin C (turquoise circle), group supplemented with tBuOOH for 24h (red triangle down), group supplemented with vitamin C and tBuOOH for 24h (magenta triangle up), group supplemented with tBuOOH for 48h (orange star), group. supplemented with vitamin C and tBuOOH for 48h (violet diamond).

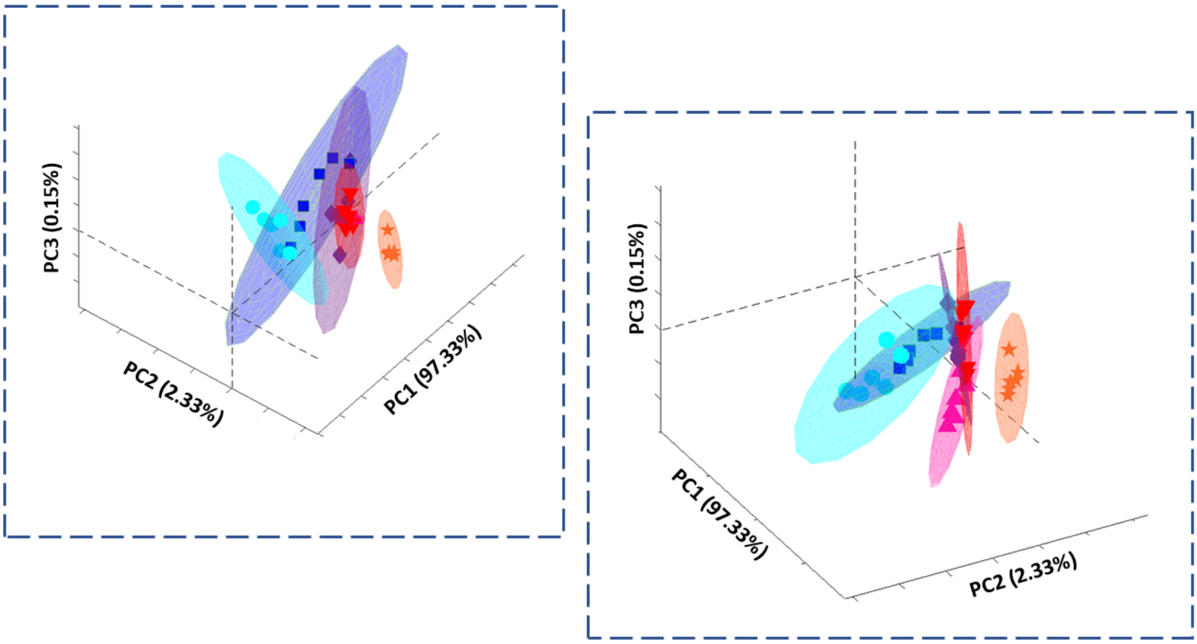

Figure S3. The scores 3D plot obtained from PCA (Principle Component Analysis) in two different projections for 6 groups of human normal colon cells: control group (blue square), group of cells supplemented with vitamin C (turquoise circle), group of cells supplemented with tBuOOH for 24h (red triangle down), group of cells supplemented with vitamin C and tBuOOH for 24h (magenta triangle up), group of cells supplemented with tBuOOH for 48h (orange star), group of cells supplemented with vitamin C and tBuOOH for 48h (violet diamond).
